# The BIG 2.04 MRC/EORTC SUPREMO Trial: pathology quality assurance of a large phase 3 randomised international clinical trial of postmastectomy radiotherapy in intermediate-risk breast cancer

**DOI:** 10.1007/s10549-017-4145-4

**Published:** 2017-02-11

**Authors:** J. S. Thomas, A. M. Hanby, N. Russell, G. van Tienhoven, K. Riddle, N. Anderson, D. A. Cameron, J. M. S. Bartlett, T. Piper, C. Cunningham, P. Canney, I. H. Kunkler, On behalf of the SUPREMO Trial Management Group

**Affiliations:** 1 Department of Pathology, Western General Hospital, Edinburgh, EH4 2XU UK; 2 Leeds Institute of Cancer and Pathology, St James’s University Hospital, Leeds, LS9 7TF UK; 3 Department of Radiation Oncology, Netherlands Cancer Institute, Postbus 90203, 1006 BE Amsterdam, Netherlands; 4 Academic Medical Center, University of Amsterdam, 1105 AZ Amsterdam, Netherlands; 5 Scottish Clinical Trials Research Unit, NHS National Services Scotland, Edinburgh, EH12 9EB UK; 6 Centre of Population Health Sciences, Edinburgh University Medical School, Edinburgh, EH8 9AG UK; 7 Edinburgh Cancer Centre, Western General Hospital, Edinburgh, EH4 2XU UK; 8 Ontario Institute for Cancer Research, Toronto, ON M5G0A3 Canada; 9 Beatson Oncology Centre, Gartnavel Campus, Glasgow, G12 0YN UK

**Keywords:** Breast cancer, Radiation therapy, Clinical trial, Pathology, Quality assurance

## Abstract

**Introduction:**

SUPREMO is a phase 3 randomised trial evaluating radiotherapy post-mastectomy for intermediate-risk breast cancer. 1688 patients were enrolled from 16 countries between 2006 and 2013. We report the results of central pathology review carried out for quality assurance.

**Patients and methods:**

A single recut haematoxylin and eosin (H&E) tumour section was assessed by one of two reviewing pathologists, blinded to the originally reported pathology and patient data. Tumour type, grade and lymphovascular invasion were reviewed to assess if they met the inclusion criteria. Slides from potentially ineligible patients on central review were scanned and reviewed online together by the two pathologists and a consensus reached. A subset of 25 of these cases was double-reported independently by the pathologists prior to the online assessment.

**Results:**

The major contributors to the trial were the UK (75%) and the Netherlands (10%). There is a striking difference in lymphovascular invasion (LVi) rates (41.6 vs. 15.1% (UK); *p* = <0.0001) and proportions of grade 3 carcinomas (54.0 vs. 42.0% (UK); *p* = <0.0001) on comparing local reporting with central review. There was no difference in the locally reported frequency of LVi rates in node-positive (N+) and node-negative (N−) subgroups (40.3 vs. 38.0%; *p* = 0.40) but a significant difference in the reviewed frequency (16.9 vs. 9.9%; *p* = 0.004). Of the N− cases, 104 (25.1%) would have been ineligible by initial central review by virtue of grade and/or lymphovascular invasion status. Following online consensus review, this fell to 70 cases (16.3% of N− cases, 4.1% of all cases).

**Conclusions:**

These data have important implications for the design, powering and interpretation of outcomes from this and future clinical trials. If critical pathology criteria are determinants for trial entry, serious consideration should be given to up-front central pathology review.

**Electronic supplementary material:**

The online version of this article (doi:10.1007/s10549-017-4145-4) contains supplementary material, which is available to authorized users.

## Introduction

BIG 2.04 SUPREMO is a phase III international MRC/EORTC randomised trial evaluating post-mastectomy radiotherapy for intermediate-risk breast cancer accruing 1688 patients from 16 countries between 2006 and 2013. Intermediate risk was defined as either node-positive (N+) (pN1) disease of any grade in tumours ≤5 cm diameter (T1 or T2), or T2 node-negative (N−) tumours that were either grade 3 and/or showed lymphovascular invasion (LVi), or T3N0 tumours, independent of pathological features. Trial entry was determined locally based on local pathological evaluation. Central pathology review was planned to be carried out later for quality assurance and not to confirm or reject trial entrants, retrospectively. This policy was adopted to allow applicability of the results to the real-world situation of daily clinical practice. To the best of our knowledge, this is the first and largest report of pathology quality assurance within an international randomised breast radiotherapy trial recruiting across three continents (Europe, Asia and Australasia). We report the results of the pathology review.

## Methods

### Patient data and pathology materials

All patient data including locally reported pathology were recorded and held centrally in the SUPREMO Trial Office at the Scottish Clinical Trials Research Unit (SCTRU), NHS Scotland in Edinburgh, UK. If multiple operations had been performed, all reports were obtained. A requirement for trial entry was the submission of a representative haematoxylin and eosin (H&E) stained section of the tumour or a paraffin block from which an H&E could be made centrally. For patients treated with neo-adjuvant systemic therapy, the initial pre-treatment biopsy tissue was used. Because of local tissue governance regulations central pathology review was restricted to hospitals from France, Germany, Japan, the Netherlands, Poland, Switzerland, Spain, Turkey, the UK, and one centre each in Australia, China and New Zealand.

### Central pathology review

The two reviewing pathologists (JT & AH) were sent in batches of 25, a single anonymised H&E section for each patient identified by the SUPREMO Trial Number only. The H&E section was usually recut rather than an original because the majority of patients had also consented to future translational studies. Data were recorded as follows: tumour type; histological grade (Bloom and Richardson as modified by Elston and Ellis 1991) [1]; and presence or absence of lymphovascular invasion (LVi). Reviewing pathologists were blinded to all patient data including locally reported pathology and node status. The pathologists are specialist breast pathologists working in large UK centres (Edinburgh and Leeds). The reviewing pathologists reported LVi according to UK reporting guidelines [2].

### Pathology quality assurance

Data were analysed as follows:

1. Completeness of data.
2. Differences between reporting profiles of reviewing pathologists and local reporting.
3. Discrepancies between local pathology reporting and central review.
  Analysis was limited to those discrepancies which would have changed a patient’s eligibility to enter the trial, i.e. a difference of overall grade or LVi which was critical to the inclusion of patients in the N− group.
  The original H&E section from the discrepant cases which had been reviewed previously by one of the pathologists was scanned at ×40 magnification using the Aperio ScanScope slide scanner (Aperio Technologies, Vista, CA) and was then viewed on line by both pathologists simultaneously, and a consensus was reached re grade and LVi. The pathologists were blinded to their original diagnoses.
4. Comparison of Nottingham Prognostic Index (NPI) in N+ and N− subgroups.
  The NPI for the two subgroups was calculated from the tumour size and number of positive nodes as reported locally and the histological grade [3]. Two calculations were made using the reported grade and the grade from central review.

### Statistical analysis

Comparison of proportions was made using a Chi squared test. Groups were compared using the Mann–Witney *U* Test. A two-sided *p* value of <0.05 was deemed significant. Statistical calculations and charts were made with Analyse-it ® v2.11 for Excel ®.

## Results

### Completeness of data

**Fig. 1:**
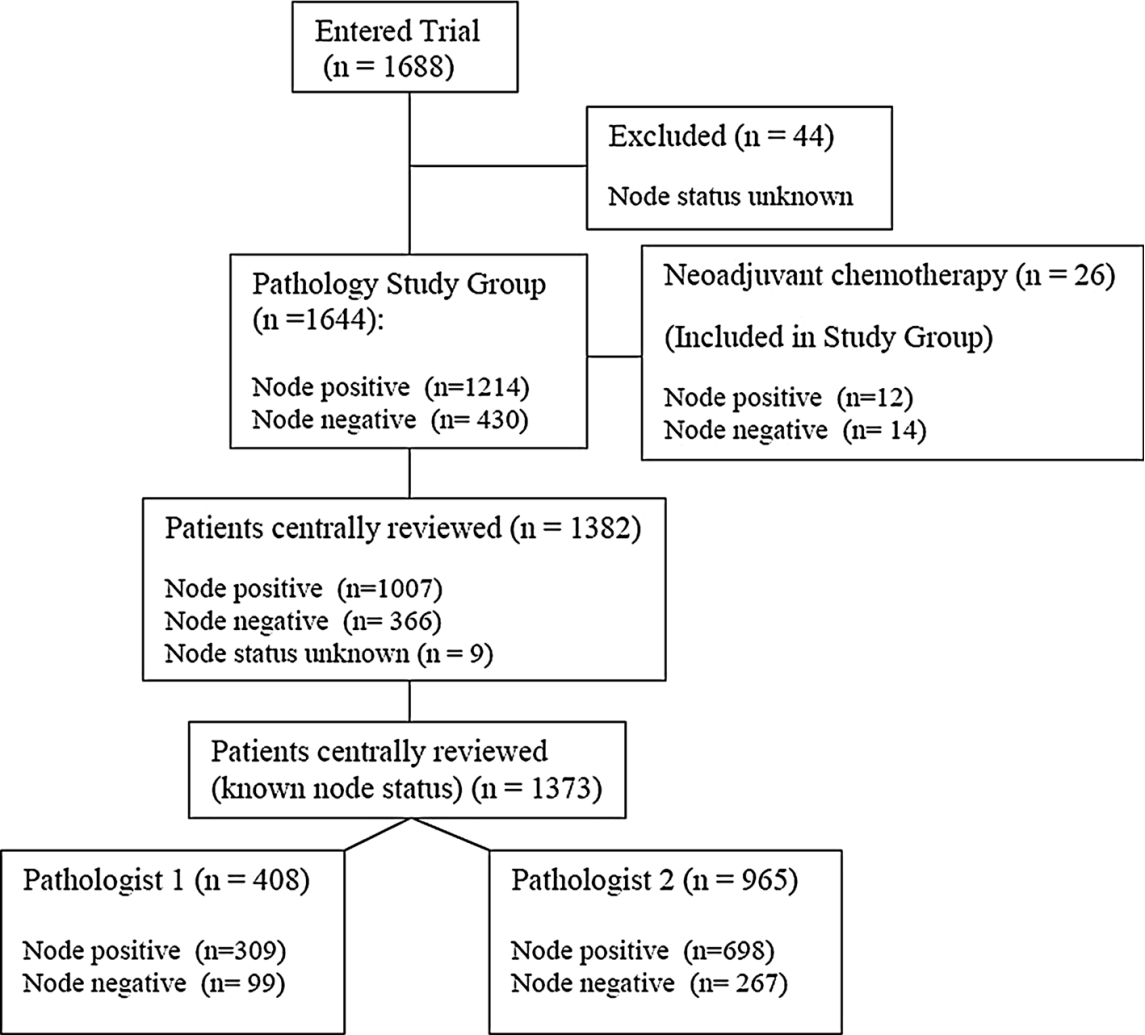
Flow diagram for Pathology QA for SUPREMO Trial

Patient enrolment and exclusions from this QA study are summarised in Fig. 1. 1688 patients were enrolled in the trial, and 44 patients were of unknown nodal status at the time of this analysis and were excluded from this study.

### Primary systemic chemotherapy patients

26 patients were treated with primary systemic chemotherapy, 12 N+ and 14 N−. The primary systemic chemotherapy patients were included in the study group. A separate analysis of the study group with the 26 primary systemic patients excluded shows no significant difference in proportions of grade 3 cases or LVi.

Reporting profiles by nationality of treating site, of reviewing pathologists and differences between central and local reporting:

The data relating to nationality of treating site are summarised in Table 1. This is limited to the top 7 (of 16) countries submitting patients accounting for 97% of the trial population. The two major contributors were the UK (75%) and the Netherlands (10%). The presence of LVi was reported locally in 41.6% of UK cases and 28.2% of Dutch cases. The difference is significant (*p* = <0.001). On central review, the frequency of LVi in the two countries was 15.1 and 19.2%, respectively. The difference is not significant (*p* = 0.23). There were 161 different randomising centres individually submitting between 1 and 70 cases (median 7 cases).

**Table 1:** Reporting profiles by country of trial entry

	No	Reviewed	%	Node Pos %	Reported		Reviewed	
					Grade 3 (%)	LVi (%)	Grade 3 (%)	LVi (%)
UK	1248	1064	85.3	73.0	54.6	41.2	42.4	15.1
Netherlands	175	154	88.0	76.0	52.0	28.4	39.6	19.5
China	60	24	40.0	100.0	26.8	31.7	45.0	28.6
France	49	35	71.4	71.4	42.8	28.5	46.7	0.0
Spain	39	36	92.3	87.1	28.2	26.3	53.8	16.7
Australia	22	17	77.3	54.5	59.1	45.4	56.2	6.2
Poland	20	18	90.0	65.0	28.6	46.1	27.8	0.0
Others (9 countries)	56	34	60.7	80.4	49.0	40.0	31.6	5.0
Total	1669	1382	82.8	74.6	52.7	39.3	41.9	15.1

The overall data relating to the N+ and N− subgroups is summarised in Table 2. There were 1214 N+ patients and 430 N− patients. The LVi rate as reported locally was high in both N+ and N− groups (39.6 vs 38.2%) and showed no significant difference (*p* = 0.40). Following central review, however, LVi was significantly different for the two groups (16.9 vs. 9.9%) (*p* = 0.004). 58 of the 708 patients who were reported locally as not showing LVi were shown to have LVi on central review (8.2%). There were similar significant differences between the overall frequency of grade 3 carcinomas as locally reported (52.7%) compared with central review (41.9%) (*p* = 0.003).

**Table 2:** Reporting of Grade 3 carcinomas and LVi by reviewing pathologists and locally in N+ and N− subgroups

	Node positive				Node negative			
	Grade 3		Lvi		Grade 3		Lvi	
	Reported	Reviewed	Reported	Reviewed	Reported	Reviewed	Reported	Reviewed
Number	496	326	481	162	362	219	158	35
%	40.8%	33.7%	39.6%	16.9%	87.4%	64.2%	38.2%	9.9%

Of the 1688 patients entered into the trial, 1382 had an H&E section available for review. The two reviewing pathologists evaluated 409 and 973 sections, respectively. The centrally reported grade and LVi profile for the two pathologists and as reported locally are summarised in Table 3. The two reviewing pathologists show similar reporting profiles, and there is no evidence of case selection bias between the two reviewed subsets.

**Table 3:** Overall reporting profile of the two reviewing pathologists and local reporting by Grade, tumour type and LVi

	Grade (No/%)						Type						
	1	%	2	%	3	%	NST	%	Lobular	%	Other	%	Lymphatic Invasion? Y (%)
Path 1	63	15.5	162	39.9	140	34.5	248	66	21	5.6	87	23.3	51 (14.2)
Path 2	57	5.8	479	49.7	409	42.5	857	89	61	6.3	45	4.7	147 (15.5)
Local	103	6.2	650	38.9	858	51.4	1155	87	91	6.8	89	6.7	639 (38.3)

A detailed breakdown of reviewed and reported LVi for all patients, and those cases reviewed centrally against both reported and reviewed grade is shown in Supplementary Tables 1a and 1b, respectively. There is a striking difference in LVi rates on comparing local reporting with central review across all grade groups.

### Prognostic equivalence of N+ and N− subgroups

**Fig. 2:**
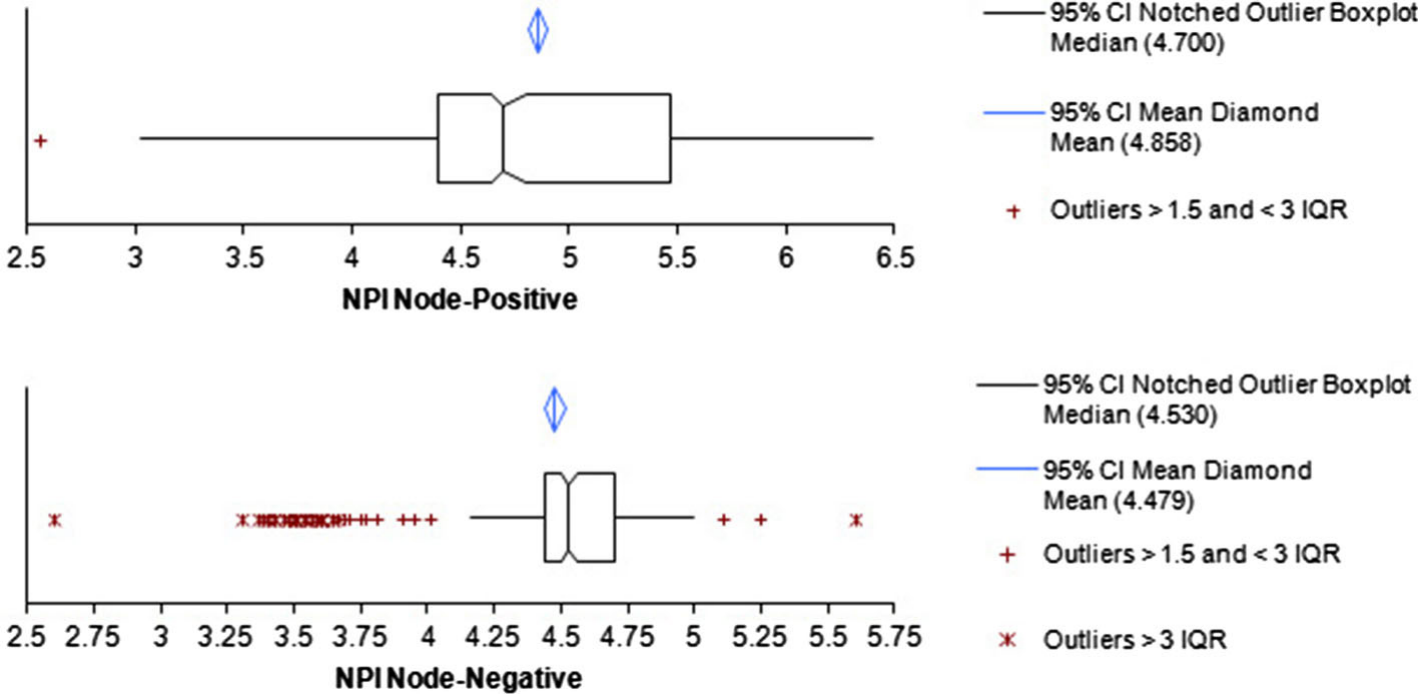
*Box plots* showing distribution of NPI scores for the N+ and N− subgroup as originally reported

The NPIs as calculated from reported grades for N+ and N− subgroups are shown in the box plots Fig. 2, and the data for reported and reviewed subgroups are summarised in Supplementary Table 2. Both the reported and reviewed NPIs in the N+ subgroup are significantly higher than the N− subgroups (both *p* = <0.0001) with large numbers of cases in the poor prognosis (NPI > 5.4) group [30 and 23%, respectively (N+) compared with <1% (N−)]. The reported and reviewed NPIs in the N− subgroup fall almost entirely (98 and 95%, respectively) within the intermediate prognostic range (NPI 3.4–5.4).

In both the N+ and N− subgroups, the mean NPI was significantly lower following review [4.70 vs. 4.60 (N+) and 4.53 vs. 4.48 (N−)] (*p* = <0.0003 and < 0.0001, respectively).

### Numbers of discrepant cases

Because pathology criteria were used to determine eligibility for the N− group, potentially ineligible cases inevitably fell in this group following a pathology QA exercise on the grounds of neither being grade 3 nor showing LVi (114 cases, 95 from the UK).

Numbers of cases per reviewing pathologist and reasons for discrepancy:

- Pathologist 1—29/409 (7%) cases: 14 cases LVi; 12 cases grade; 3 cases both
- Pathologist 2—85/873 (10%) cases: 33 cases LVi; 47 cases Grade; 15 cases both

Of these 114 cases, 108 were scanned satisfactorily and were available for review online by the two pathologists. 23 cases were upgraded on review from grade 2 to 3, and a further 12 cases were agreed to show LVi. Therefore, 32% of cases originally deemed ineligible by initial central review were deemed eligible following joint discussion.

### Cross-over reporting

25 cases were re-reported from slides by the two pathologists independently. There was complete agreement on grade in 20 cases (80%). 5 cases showed grade 2/3 disagreements (20%). There was no evidence of grade bias by either pathologist. 2 cases showed disagreement about LVi (8%).

## Discussion

### Implications for patient eligibility for SUPREMO and other clinical trials

Following a central review of pathology variables in the SUPREMO Trial population, we identified 19% of N− patients who would, if central pathology data were used, be ineligible for the trial. Whilst the total number of cases deemed ineligible by central review was low, it represents a significant sub-group of the N− patients.

The non-eligible rate for our N− subgroup raises concerns about the interpretation of outcomes from this trial, particularly in the N− subgroup. Our data raise questions about whether clinical trials need to be powered to accommodate significant minorities of patients actually being ineligible or should they reflect practice in the real world? In the ARTemis trial, the principal pathological end point was confirmed by review of pathology reports by the clinical investigators [4]. This was because the trial was powered on the basis of full recruitment, whereas slide retrieval was anticipated to be 85% of entrants at best. If it is decided that pathological central review is the desired way to assess a particular outcome, then the powering of the trial will need to be adjusted to allow for this estimated retrieval rate of around 85%.

In the SUPREMO trial, N− patients were required to have either grade 3 carcinomas or LVi or both, whereas N+ patients were not. This was an attempt to ensure a degree of prognostic equivalence between the two groups. We compared the two groups looking at their respective NPIs to test this assumption and found a significant difference between them. We appreciate that the NPI does not include LVi as a factor and so this tool only examined this issue partially.

### Critical evaluation of this central pathology review

The following issues need to be considered in the interpretation of our data:

1. We reviewed a single recut H&E section and not the original tumour sections available to the local pathologists. We accept fully that this will lead inevitably to a lower reviewed LVi frequency compared with the local frequency. The availability of a single H&E for central review is certainly an important issue in explaining the lower LVi frequency on central review but does not explain the lack of difference in local reporting between N+ and N− subgroups.
2. In the original trial protocol, specific instructions were not given as to how LVi should be reported. The reviewing pathologists did not meet to discuss how this aspect of the review should be carried out but simply followed the UK guidelines as per their normal practice. In view of fact that 75% of SUPREMO cases were from the UK, we would expect these cases to have been reported according to standard UK practice. It is notable that SUPREMO Trial cases were not entered into the trial until the MDM where the case was discussed—therefore *after* it had been reported. It follows that on average in the UK patients with intermediate-risk breast cancer (whether N+ or N−) have an LVi frequency of >40%. This is not in line with the reviewing pathologists’ experience.
3. When the reviewing pathologists carried out the cross-over review, they upgraded LVi status on 20% of cases. If this were extrapolated across the whole N− group (assuming that the status change was always in one direction), then the LVi frequency would rise from 15 to 19%. That is still a long way from 41%.
4. The proximity of reporting profiles of the two reviewing pathologists is remarkably close, and it is of concern that the reviewing pathologists consistently found a substantially lower rate of LVi than was locally reported where the bias was in favour of the presence of LVi rather than its absence. There is a trend in our data of increased frequency of LVi with increasing grade, but there is no difference between the frequency of reported LVi in the N+ and N− groups, whereas this was a consistent finding by the two reviewing pathologists. In the Nottingham case series, there were strong correlations between nodal status and tumour grade and LVi where 12% of grade 1 carcinomas and 40% of grade 3 carcinomas showed LVi [5]. Two further large studies of LVi in N- breast cancer have shown overall rates of 19.5 and 19%, respectively [6, 7]. In the Uppsala, radiotherapy trial for Stage 1 breast cancer where all tumour slides were reviewed LVi was recorded in 22% of cases [8].
5. Our data also show significant differences between the frequency of grade 3 carcinomas as reported locally (53%) and following central review (42%). The central review figure is very close to that reported in the Nottingham series of 3255 patients where grade 3 carcinomas accounted for 43% of cases overall [5].
6. From a logistical point of view, the QA process for this trial was labour-intensive. The two reviewing pathologists (AH & JT) are currently carrying out the pathology QA for the LORIS trial [9] where pathological eligibility criteria are confirmed at the time of diagnosis by near-real-time review of scanned images on line. Using this approach all potential patients’ pathology is turned around within five working days with no delay to the patient’s management pathway.

### Consistency of reporting among pathologists

There is substantial variability in the grading consistency of pathologists [10], although a recent study showed moderate to good consistency for grades 1 & 3 (kappa = 0.7) [11] in a large review of the NHS Breast Screening Programme EQA Scheme the kappa for grade was lower at 0.48 [12]. The literature is, however, conflicting on consistency of reporting by generalist and specialist pathologists [13–15]. It is encouraging to note that there were no major differences in the broad metrics of reporting profiles between the major countries contributing to this trial.

### Comparability of the N+ and N− subgroups

NPI has been tested extensively as a prognostic tool and has been shown to correlate well with medium and long-term outcomes [16, 17]. This trial was designed and powered on the assumption that the presence of grade 3 histology and/or lymphatic invasion would render the N− patients prognostically equivalent to those with N+ disease. This will only be known when outcome data become available when the trial reports.

## Conclusion

This international study provides unique data comparing local reporting and central review of pathology for a large clinical trial in three continents. Pathology criteria were critical for the inclusion of N− patients and central review even after arbitration suggest that up to 20% of this subgroup were ineligible for trial entry. The study raises questions about design of clinical trials, particularly how they are powered, the methodology of central pathology review and the role of digital technology in supporting this process. Consistency in pathology reporting between Europe and China provides a sound platform for collaboration in clinical trials requiring multinational accrual.

## Electronic supplementary material

Below is the link to the electronic supplementary material.

Supplementary Table 1a Numbers of cases with LVi against histological grade as locally reported for all cases and according to nodal status (DOCX 14 kb)

Supplementary Table 1b Numbers of cases with LVi against histological grade as centrally reviewed for all cases and according to nodal status (DOCX 14 kb)

Supplementary Table 2 Distribution of NPI scores for N+ and N− subgroups as reported locally and on central review (DOCX 10 kb)
