# Supplementary material for: The BIG 2.04 MRC/EORTC SUPREMO Trial: pathology quality assurance of a large phase 3 randomised international clinical trial of postmastectomy radiotherapy in intermediate-risk breast cancer
Source: Breast Cancer Res Treat. 2017 Feb 11;163(1):63–9. doi: 10.1007/s10549-017-4145-4 (PMC5387007; doi:10.1007/s10549-017-4145-4)
Supplement: Supplementary file 1 — Numbers of cases with LVi against histological grade as locally reported for all cases and according to nodal status (DOCX 14 kb) [file 10549_2017_4145_MOESM1_ESM.docx]

Supplementary Table 1a

|  | Reviewed | All cases | | | | Pathologist 1 | | Pathologist 1 | | Pathologist 2 | | Pathologist 2 | |
| --- | --- | --- | --- | --- | --- | --- | --- | --- | --- | --- | --- | --- | --- |
|  | Grade | Reported Lvi | | Reviewed Lvi | | Reported Lvi | | Reviewed Lvi | | Reported Lvi | | Reviewed Lvi | |
|  |  | No | % | No | % | No | % | No | % | No | % | No | % |
| All | 1 | 46 | 38.33% | 6 | 5.00% | 22 | 34.92% | 5 | 7.94% | 24 | 42.11% | 1 | 1.75% |
| Cases | 2 | 250 | 39.00% | 116 | 18.13% | 67 | 41.36% | 23 | 14.29% | 183 | 38.20% | 93 | 19.42% |
|  | 3 | 226 | 41.17% | 76 | 13.89% | 63 | 45.00% | 23 | 16.67% | 163 | 39.85% | 53 | 12.96% |
| Node | 1 | 38 | 34.55% | 6 | 5.50% | 20 | 32.79% | 5 | 8.20% | 18 | 36.73% | 1 | 2.08% |
| Positive | 2 | 195 | 37.14% | 99 | 19.00% | 51 | 38.35% | 19 | 14.73% | 144 | 36.73% | 80 | 20.41% |
|  | 3 | 157 | 48.16% | 57 | 17.65% | 39 | 46.43% | 14 | 17.28% | 118 | 48.76% | 43 | 17.77% |
| Node | 1 | 8 | 80.00% | 0 | 0.00% | 2 | 100.00% | 0 | 0.00% | 6 | 75.00% | 0 | 0.00% |
| Negative | 2 | 55 | 49.11% | 17 | 15.32% | 16 | 55.17% | 4 | 14.29% | 39 | 46.99% | 13 | 15.66% |
|  | 3 | 69 | 31.51% | 18 | 8.22% | 24 | 42.86% | 9 | 16.07% | 45 | 27.61% | 9 | 5.52% |
