# Supplementary material for: The BIG 2.04 MRC/EORTC SUPREMO Trial: pathology quality assurance of a large phase 3 randomised international clinical trial of postmastectomy radiotherapy in intermediate-risk breast cancer
Source: Breast Cancer Res Treat. 2017 Feb 11;163(1):63–9. doi: 10.1007/s10549-017-4145-4 (PMC5387007; doi:10.1007/s10549-017-4145-4)
Supplement: Supplementary file 2 — Numbers of cases with LVi against histological grade as centrally reviewed for all cases and according to nodal status (DOCX 14 kb) [file 10549_2017_4145_MOESM2_ESM.docx]

Supplementary Table 1b

|  | Reported | All Cases | | | | Pathologist 1 | | Pathologist 1 | | Pathologist 2 | | Pathologist 2 | |
| --- | --- | --- | --- | --- | --- | --- | --- | --- | --- | --- | --- | --- | --- |
|  | Grade | Reported Lvi | | Reviewed Lvi | | Reported Lvi | | Reviewed Lvi | | Reported Lvi | | Reviewed Lvi | |
|  |  | No | % | No | % | No | % | No | % | No | % | No | % |
| All | 1 | 22 | 21.36% | 2 | 2.86% | 10 | 29.41% | 1 | 3.33% | 7 | 15.91% | 1 | 2.50% |
| Cases | 2 | 240 | 36.92% | 85 | 16.47% | 56 | 34.57% | 18 | 12.77% | 148 | 39.05% | 67 | 17.87% |
|  | 3 | 377 | 43.94% | 109 | 15.53% | 102 | 49.04% | 32 | 17.11% | 221 | 42.34% | 77 | 14.95% |
| Node | 1 | 19 | 19.00% | 2 | 2.94% | 8 | 25.00% | 1 | 3.57% | 7 | 15.91% | 1 | 2.50% |
| Positive | 2 | 198 | 32.62% | 79 | 16.36% | 48 | 31.17% | 16 | 11.85% | 120 | 34.19% | 63 | 18.10% |
|  | 3 | 264 | 53.23% | 80 | 19.95% | 66 | 54.10% | 21 | 19.44% | 158 | 52.84% | 59 | 20.14% |
| Node | 1 | 3 | 100.00% | 0 | 0.00% | 2 | 100.00% | 0 | 0.00% | 0 | 0.00% | 0 | 0.00% |
| Negative | 2 | 42 | 97.67% | 6 | 18.18% | 8 | 100.00% | 2 | 33.33% | 28 | 100.00% | 4 | 14.81% |
|  | 3 | 113 | 31.22% | 29 | 9.63% | 36 | 41.86% | 11 | 13.92% | 63 | 28.25% | 18 | 8.11% |
