# Supplementary material for: The BIG 2.04 MRC/EORTC SUPREMO Trial: pathology quality assurance of a large phase 3 randomised international clinical trial of postmastectomy radiotherapy in intermediate-risk breast cancer
Source: Breast Cancer Res Treat. 2017 Feb 11;163(1):63–9. doi: 10.1007/s10549-017-4145-4 (PMC5387007; doi:10.1007/s10549-017-4145-4)
Supplement: Supplementary file 3 — Distribution of NPI scores for N+ and N− subgroups as reported locally and on central review (DOCX 10 kb) [file 10549_2017_4145_MOESM3_ESM.docx]

Supplementary Table 2

|  | *n* | Median | IQR | 95% CI |
| --- | --- | --- | --- | --- |
| N+ Reported NPI | 994 | 4.70 | 1.06 | 4.64-4.80 |
| N- Reported NPI | 362 | 4.53 | 0.26 | 4.50-4.56 |
| N+ Reviewed NPI | 784 | 4.60 | 1.00 | 4.56-4.62 |
| N- Reviewed NPI | 302 | 4.48 | 0.98 | 4.40-4.50 |
